# Videos and Vocabulary: How Digital Media Use Impacts the Types of Words Children Know

**DOI:** 10.1111/desc.70091

**Published:** 2025-11-10

**Authors:** Sarah C. Kucker, Rachel F. Barr, Lynn K. Perry

**Affiliations:** ^1^ Southern Methodist University Dallas Texas USA; ^2^ Georgetown University Washington District of Columbia USA; ^3^ University of Miami Coral Gables Florida USA

**Keywords:** digital media, individual differences, infant/toddler, multimodal learning, vocabulary development

## Abstract

The last decade has seen an exponential rise in children's digital media use, as well as growing evidence that it is associated with changes in children's vocabulary. However, while high rates of low‐quality digital media have been associated with lower *amounts* of words a child says, little work has examined whether digital media alters *the types* of words a child knows. Here, we explore whether differences in the amount of digital media exposure are associated with differences in the composition of children's vocabulary. The current study surveyed 388 caregivers of children 17–30 months (*M* = 23.9 months) on their children's productive vocabulary and technology use. Multiple regression models predicted the proportion of words children knew in different semantic categories based on the time they spent watching videos/TV, controlling for total noun vocabulary size, age, and income. Increased video watching was associated with producing a smaller proportion of body part words and more people and furniture words, but not other semantic categories. Increased video watching was not associated with differences in shape‐ or material‐based nouns. The results suggest that differences in children's video watching are associated with differences in overall vocabulary size, but also with the particular types of words children know. This may have implications for supporting children's future language in a technology‐filled world.

**Summary:**

- Digital media exposure has been associated with changes in children's vocabularies. However, little work examines whether the media alters the *types* of words a child learns.
- The current study explores whether differences in the amount of digital media exposure are associated with differences in the composition of children's vocabulary.
- Increased video watching was associated with producing fewer body part words, more people, and more furniture words, but no difference in shape‐ or material‐based nouns.
- The results suggest that differences in children's video watching are associated with differences in overall vocabulary size, but also with the types of words children know.

## 1 Introduction

The last decade has seen an exponential rise in the amount of digital media that young children consume. Current estimates find children under 2 years spend 2 h per day watching videos/TV and approximately 15 min each in video chat, games/apps, and e‐books (Kucker et al. 2024; Mann et al. 2025). Some of these forms of media can be beneficial for children and help language development—for example, high‐quality educational apps (Jing et al. 2023) and co‐viewing (Strouse et al. 2018). However, there is also evidence that the use of low‐quality videos/TV results in fewer social interactions with caregivers (Anderson and Hanson 2017; Brushe et al. 2024; Christakis et al. 2009), less active play (Putnick et al. 2023), and less tactile exploration (Ziemer and Snyder 2016) compared to when digital media is not present. Social, active, and multi‐modal experiences are critical parts of a child's language learning environment, supporting broad vocabulary growth during the first 2 years of life, but also learning for specific types of words. Recent research suggests that lower quality media that is missing interactive and social elements is sometimes associated with a lower *overall amount* of words being learned (Kucker et al. 2024; Madigan et al. 2020; Sundqvist et al. 2024; but see Jing et al. 2023, Taylor et al. 2018). However, because digital media reduces opportunities for specific types of social interactions and multi‐modal exploration, it may change *the types* of words a child learns. Here, in a large sample of 17–30‐month‐old children, we examine associations between children's exposure to videos/TV and the types of words they know.

### 1.1 Digital Media Exposure and Effects on Children's Experiences

Over the last decade, children have been increasingly exposed to digital media, with measurable exposure starting before they begin to talk (Mann et al. 2025). This exposure changes children's daily experiences and the ways in which they engage with the world, especially relating to language. First, as children's time watching videos increases, they spend less time interacting with people and objects (Anderson and Hanson 2017; Christakis et al. 2009). As they engage with fewer people, they hear less speech directed at them and produce fewer utterances themselves (Brushe et al. 2024). This means children get less language input from social partners or face‐to‐face social interactions. This lack of naturalistic social input is not necessarily replaced by digital content either. A recent corpus analysis by Kolak et al. (2023) found that child‐based educational apps had less lexical diversity and shorter utterances than everyday face‐to‐face language input. Wong, Neuman, and colleagues (Danielson et al. 2019; Wong and Neuman 2019) similarly discovered that many educational videos use overly simplistic vocabulary centered on labeling concrete objects. Teaching of new words was limited to ostensive naming and repetition, which helps learning, but is less natural and less rich than real‐world input.

Second, digital media (and especially videos/TV) change how children interact and engage with content. When children watch videos, they are limited in the modalities by which they can physically engage (i.e., videos present primarily 2D images without tactile, olfactory, or gustatory input). Many current forms of digital media are less social, less embodied, and contain simplified features. Work by Zeimer and colleagues (Ziemer et al. 2021; Ziemer and Snyder 2016) has demonstrated that the ways in which children haptically explore images on a tablet vary from interactions with real physical objects or picture books—actions toward digital media are much more simplistic and less object‐specific. Children's interactions with digital media are less *multi*‐modal and less object‐oriented, limiting information such as 3D shape and material that are helpful for learning object labels (Schroer and Yu 2022; Slone et al. 2019).

Thus, digital media exposure likely changes specific opportunities children have for learning in the world. Namely, (1) it decreases opportunities for language learning in face‐to‐face social interactions and (2) affords a different sensory experience and unique content from what children would otherwise encounter in the physical world. These differences are critical as both social interactions and multimodal exploration impact children's language development. On the one hand, when videos are well‐designed to contain language‐promoting elements, especially narrative structure, they can promote vocabulary development (Linebarger et al. 2017; Linebarger and Vaala 2010; Vaala et al. 2010). For example, in a longitudinal study of 6–30‐month‐olds, well‐designed educational content was associated with a larger vocabulary, and low‐quality content was associated with a smaller vocabulary size (Linebarger and Walker 2005). Likewise, when digital media is used with a social partner, or the form of media requires joint engagement, such as video chat, thereby mirroring typical language experiences, there is less of a detriment to vocabulary (Jing et al. 2023; Strouse et al. 2018).

On the other hand, some evidence suggests that as time spent viewing low‐quality videos/TV rises, children's overall vocabularies decrease (Kucker et al. 2024; Madigan et al. 2020). Children also struggle to learn new words when presented in a digital modality (Strouse and Samson 2021). But, decreases in vocabulary due to digital media use have been found to be fully mediated by the quality and quantity of caregiver talk (Sundqvist et al. 2022), reinforcing the importance of social interactions. This all means the rising use of digital media changes children's language learning environments in ways that may impact the acquisition of early learned words, especially those relying on social and multimodal input.

### 1.2 Early Vocabulary Composition and the Types of Words Children Learn

From 18–30 months, a child's vocabulary rapidly grows from fewer than 100 words to more than 500 (Fenson et al. 1994; Frank et al. 2017). This growth is strengthened by social and multi‐modal experiences (Custode and Tamis‐LeMonda 2020; Tamis‐LeMonda and Bornstein 1993). Social and multi‐modal exploration helps vocabulary in general, but social interactions also have particular utility for learning specific semantic classes of words such as names for people (mommy, daddy; Tincoff and Jusczyk 1999) and body parts (hand, nose; Bergelson and Swingley 2012; Tincoff and Jusczyk 2012). During dyadic interactions, words for people are highly frequent (Goodman et al. 2008; Swingley and Humphrey 2018), making it easier for children to learn such words. Caregivers also touch and socially refer to the child's body while labeling these body parts; children who experience such behavior learn those terms more readily (Seidl et al. 2015; Tincoff et al. 2019). As a result, people and body parts are often some of the earliest learned classes of words (Bergelson and Swingley 2012; Frank et al. 2017).

Additionally, multi‐modal exploration helps children's learning for types of words with specific features, such as shape or material. Easily manipulatable items are some of the earliest learned words (ball, cup; Bergelson and Aslin 2017; Frank et al. 2017), and when children have an opportunity to explore and manipulate 3D items, they are more likely to learn the item's name and features (Kucker and Samuelson 2012; Smith 2005; Suarez‐Rivera et al. 2022). Physically exploring these objects helps children see the 3D shape, which in turn helps with learning and vocabulary growth (Slone et al. 2019). The impact of multi‐modal exploration is also beneficial for learning names for categories organized by similarity in material (e.g., chalk), including non‐solid substances (applesauce; Perry et al. 2014), as tactile information is generally needed to recognize and classify materials (Lederman and Klatzky 1990). For instance, children who engage in more “messy” whole‐handed manual exploration are better able to identify and generalize object labels compared to children who merely look at or gently poke stimuli with a single finger (Perry et al. 2014). The ability to haptically explore may be essential for learning specific words where either shape or material is the defining feature.

Overall, opportunities for social and multi‐modal experiences, which are often diminished in digital media relative to other contexts, are key for learning specific classes of words. But why might the types of words matter above and beyond total vocabulary size? The types of words a child learns early are critical for future vocabulary growth. For instance, children who have an early, robust knowledge of highly familiar words, including their own name and other people words, can use these words as a foundation for segmenting and recognizing novel speech (Bortfeld et al. 2005). Children who know clusters of semantically similar words tend to have stronger word learning abilities (Borovsky et al. 2016a; Borovsky and Elman 2006) and faster vocabulary growth (Borovsky 2022); one common cluster of words for many children is body parts (Borovsky et al. 2016b). Likewise, many early learned object nouns refer to categories organized by similarity in shape, and children who know a higher proportion of these nouns show reliable generalization (Gershkoff‐Stowe and Smith 2004; Perry and Samuelson 2011) as well as better long‐term vocabulary outcomes (Perry et al. 2023). Children whose vocabularies do not show these same structures often have persisting language delays (MacRoy‐Higgins et al. 2016; Perry et al. 2023). Children who know fewer nouns (Colunga and Sims 2017; MacRoy‐Higgins et al. 2016) or know fewer clusters of semantically‐related words and more odd‐ball words (e.g., less likely to know arm/hand than milk/pool; Beckage et al. 2011) are more likely to be “late talkers.” This suggests that examining the semantic classes of words may be insightful for growth. Additionally, toddlers whose vocabularies are not dominated by shape‐based nouns are more likely to receive a Developmental Language Disorder (DLD) diagnosis 4–7 years later (Perry et al. 2023). Thus, the composition of a child's vocabulary is arguably as important to study as overall vocabulary size, as it could be useful for predicting future language growth. This is particularly true when the landscape of children's language learning is increasingly including digital contexts.

### 1.3 Current Study

The rising prevalence of digital media at a key time in children's language development means understanding digital media's precise impact on vocabulary is crucial. Despite work showing correlations between digital media use and overall vocabulary, however, little research examines differences in the specific types of words children learn. Because digital media changes opportunities for social interactions and multi‐modal exploration, and such experiences are helpful for learning specific types of words, digital media use may also be associated with differences in a child's vocabulary composition. The current study seeks to fill this gap with a large sample of children in the midst of the vocabulary spurt. We specifically examine differences in children's semantic categories of words as well as differences in their shape and material‐based noun vocabularies. Although we pay particular attention to people's words and body part words due to their role in early vocabulary acquisition, the analyses of semantic classes are considered exploratory due to the lack of prior work exploring knowledge of other semantic categories. The analysis on shape‐ and material‐words was based on prior work (Perry et al. 2023; Samuelson and Smith 1999) and was pre‐registered with a hypothesis that children with higher media use would have fewer shape‐based nouns in their vocabulary. In line with this, we hypothesized that higher digital media use would also result in children knowing fewer material‐based words.

## 2 Methods

### 2.1 Participants

A total of 388 caregivers of children 17–30 months (*M* = 23.9 months) participated. Individuals were recruited through CloudResearch from February 2022 to May 2023 (*n* = 268) or in‐person while they participated in a study on word learning from June 2022 to July 2024 (*n* = 120). All responses were cleaned and validated prior to analysis per recommendations (Chmielewski & Kucker 2020). An additional 67 caregivers from the online sample completed the study but were not included due to failing validity checks (*n* = 55), being bilingual (*n* = 10), or having children with significant diagnosed delays (*n* = 2). The online sample reported similar age and racial and gender distribution, but slightly lower vocabulary abilities and caregiver education/income compared to the in‐lab sample. See Table 1. Samples are collapsed together for analysis, but age and caregiver income are included as covariates. The digital media use rates and motivations for digital media use for most of the online portion of the sample are reported in Kucker et al. (2024); vocabulary composition data were not analyzed previously.

**TABLE 1:** Demographic information.

	Lab sample (*n* = 120)			Online sample (*n* = 268)			Between groups
	*M*	SD	Range	*M*	SD	Range	
**Child age (months; days)**	24;9	4;15	16;19–31;16	23;22	4;8	16;11–32;29	*t*(386) = 1.11, *p* = 0.27 [−44.24, 12.30]
**Child vocabulary**	257.13	196.32	1–654	176.79	172.94	0–664	*t*(386) = 4.05, *p* < 0.001 [−119.31, −41.36]
**Child noun vocabulary**	150.79	107.32	0–359	101.35	94.32	0–361	*t*(386) = 6.90, *p* < 0.001 [−70.75, −28.14]
**Child gender**	Male *n* = 67 Female *n* = 53 Not reported = 0			Male *n* = 146 Female *n* = 121 Not reported = 1			χ^2^(1, 387) = 0.04, *p* = 0.83
**Child Hispanic**	*n* = 19			*n* = 29			χ^2^(1, 388) = 1.92, *p* = 0.17
**Child race**	White = 96 Multiracial = 14 Black = 8 Asian = 1 Native American/American Indian = 0 Not reported = 1 Not listed = 0			White = 205 Multiracial = 24 Black = 28 Asian = 6 Native American/American Indian = 2 Not reported = 1 Not listed = 2			χ^2^(4, 388) = 3.51, *p* = 0.48
**Ave. caregiver education** ^a^	6.38	1.14	3–8	5.23	1.29	2–8	*t*(386) = 8.44, *p* < 0.001 [−1.47, −0.91]
**Caregiver annual income** ^b^	14.90	5.79	2–21	8.63	4.60	1–21	*t*(379) = 11.25, *p* < 0.001 [−7.25, −5.16]

^a^Education was averaged across both caregivers and rank ordered; less than 7^th^ grade was reported as a 1, Doctoral degree was reported as 8, 4‐year college degree was equivalent to a score of 6.

^b^Income was rank ordered in $10,000 increments. “Less than $10,000” scored as 1, up to “over $200,000” as 21.

### 2.2 Measures

Each caregiver completed questionnaires concerning their children's technology exposure and vocabulary.

#### 2.2.1 Technology Use

The media assessment questionnaire (MAQ v2.1; Barr et al. 2020; Barr, Kirkorian, Radesky et al. 2024) captured children's technology use. Caregivers reported the number of minutes their child spends on various forms of technology on a typical weekday and weekend. Because the majority of children's digital media exposure at this age is spent watching videos/TV (Kucker et al. 2024), and prior work shows time spent with videos/TV is associated with lower vocabulary (Madigan et al. 2020), the average amount of videos/TV per day is used as the primary media variable here. To calculate average daily TV viewing screen time, weekday values were weighted by 5 and weekend values by 2, the products were summed, and divided by 7 to obtain average daily minutes of media use for each child. Most children (93.8%) watched some amount of videos/TV with an average of nearly 2 h per day (*M* = 109.63 min, SD = 101.51 min; range = 0–480).

#### 2.2.2 Vocabulary Composition

Children's vocabulary was captured with the MacArthur‐Bates Communicative Development Inventory: Words & Sentences (MBCDI; Fenson et al. 1994). The average productive vocabulary was 202 words (SD = 184, range = 0–664). We measured the composition of children's noun vocabularies with respect to (1) the density of knowledge in various semantic categories and (2) dominant category feature (shape vs. material). First, for semantic categories, we calculated the proportion of children's noun vocabulary that consisted of words produced in each of the noun sections of the MCDI (sections 2–12) consistent with prior work (Borovsky 2022; Borovsky et al. 2016a, 2016b) (e.g., animals from the words in section 2, people words in section 12; see Fenson et al. 1994 for details on each section).

Second, we classified all object nouns (MCDI sections 2–10) based on their dominant category features (shape‐based vs. material‐based; see Perry et al. 2023; Samuelson and Smith 1999). Shape‐based nouns included 185 words from categories of solid objects organized by similarity in shape using count syntax, such as “a,” “one,” or “two” (e.g., ball), categories of solid objects organized by similarity in shape with no agreed upon syntax (e.g., green beans), and categories organized by similarity in shape using count syntax, but have ambiguous solidity (e.g., sweater). There are 50 “material‐based” nouns that include nonsolid substances in categories organized by similarity in material that use mass syntax, such as “some” (e.g., applesauce), categories of nonsolid substances organized by similarity in material that have no agreed upon syntax (e.g., soda/pop), categories organized by material that use mass syntax, but have ambiguous solidity (e.g., butter), categories of solid objects organized by material similarity that use mass syntax (e.g., chalk), and categories organized by material that use count syntax (e.g., towel).

### 2.3 Analytical Approach

Multiple regression models predicted the proportion of words children knew in different semantic categories based on the time they spent watching videos/TV, controlling for total noun vocabulary size, age, and caregiver income. First, we examined associations between video watching and the proportion of nouns a child produced in each section of the MCDI (sections 2–12; e.g., animals, food, body parts, people). Second, we examined associations between video watching and the proportion of nouns a child produced that named objects with shape‐based (e.g., ball, cup) versus material‐based (e.g., chalk, applesauce) category organization (see Perry and Samuelson 2011; Samuelson and Smith 1999). All variables were centered prior to analysis. A Benjamini‐Hochberg correction was applied to the *p* values of each set of analyses given the multiple, yet exploratory nature of the tests. Portions of the study were pre‐registered including data collection and initial vocabulary analysis of shape versus material‐based nouns (https://osf.io/42bgj/?view_only=90e2a307ca0943e8b31312fc9bcbc743; hypothesis 3); analyses on semantic categories were not pre‐registered. Data and analytic code are publicly accessible via OSF: https://osf.io/zyr4f/?view_only=d5b0e7bd0f004b3ea821ac78a55deb56.

## 3 Results

Children's digital media use (average daily time spent consuming videos/TV) was assessed in comparison to the composition of their vocabulary based on semantic class (MCDI sections 2–12) and noun classifications (shape‐ and material‐based words; Samuelson and Smith 1999). Overall, children's digital media time was negatively correlated with their overall vocabulary, *r* = −0.10, *p* = 0.040, 95% CI [−0.20, −0.00], total noun vocabulary, *r* = −0.13, *p* = 0.014, 95% CI [−0.22, −0.03], and caregiver income, *r* = −0.24, *p* < 0.001, 95% CI [−0.33, −0.14]. Preliminary analysis showed children's total noun vocabulary is also positively correlated with children's age, *r* = 0.53, *p* < 0.001, 95% CI [0.45, 0.59], and caregiver income, *r* = 0.23, *p* < 0.001, 95% CI [0.13, 0.32]. Thus, children's age, caregiver income, and total noun vocabulary were controlled for in each subsequent analysis.

### 3.1 Digital Media and Semantic Classes of Nouns

First, we examined associations between video watching and the proportion of nouns a child produced in each section of the MCDI (sections 2–12; e.g., animals, food, body parts, people). We found that increased video watching was associated with producing fewer body part words, more people words, and more furniture words, but not other semantic categories, *p's* > 0.05. See Table 2.

**TABLE 2:** Regression analyses predicting vocabulary knowledge in different semantic domains from video time, controlling for overall vocabulary, child age, and caregiver income.

Model	Predictor	*Β*	SE	*t*	*p_adjusted_*
Section 2—animals (real or toy)	Video time	−0.01	0.00	−1.85	0.161
	Child age	0.00	0.00	0.22	0.915
	Caregiver income	0.01	0.00	1.27	0.407
	Noun vocab (sect. 2–12)	−0.01	0.01	−1.28	0.435
Section 3—vehicles (real or toy)	Video time	−0.00	0.00	−0.95	0.580
	Child age	0.00	0.00	0.13	0.933
	Caregiver income	0.00	0.00	0.77	0.667
	Noun vocab (sect. 2–12)	−0.01	0.00	−2.40	0.068
Section 4—toys	Video time	0.00	0.00	0.27	0.909
	Child age	0.00	0.00	1.16	0.435
	Caregiver income	0.00	0.00	0.47	0.835
	Noun vocab (sect. 2–12)	−0.02	0.00	−6.83	<0.001^***^
Section 5 – food and drink	Video time	−0.01	0.00	−1.67	0.209
	Child age	0.00	0.00	0.29	0.909
	Caregiver income	0.00	0.00	0.21	0.915
	Noun vocab (sect. 2–12)	0.02	0.00	4.05	<0.001^***^
Section 6 – clothing	Video time	−0.00	0.00	−0.86	0.635
	Child age	−0.00	0.00	−0.40	0.867
	Caregiver income	0.00	0.00	0.09	0.933
	Noun vocab (sect. 2–12)	0.01	0.00	2.26	0.091
**Section 7 – body parts**	**Video time**	**−0.01**	**0.00**	**−3.21**	**0.008** ^**^
	Child age	0.01	0.00	1.77	0.181
	Caregiver income	−0.01	0.00	−1.93	0.160
	Noun vocab (sect. 2–12)	−0.01	0.00	−1.90	0.160
Section 8 – small household items	Video time	0.00	0.00	0.65	0.731
	Child age	−0.00	0.00	−0.66	0.731
	Caregiver income	−0.00	0.00	−0.46	0.835
	Noun vocab (sect. 2–12)	0.03	0.00	12.85	<0.001^***^
**Section 9 – furniture and rooms**	**Video time**	**0.01**	**0.00**	**3.15**	**0.009** ^**^
	Child age	0.00	0.00	0.10	0.933
	Caregiver income	−0.00	0.00	−2.05	0.130
	Noun vocab (sect. 2–12)	0.02	0.00	9.89	<0.001^***^
Section 10 – outside things	Video time	−0.00	0.00	−0.55	0.803
	Child age	0.00	0.00	0.84	0.635
	Caregiver income	0.00	0.00	1.62	0.222
	Noun vocab (sect. 2–12)	0.02	0.00	8.20	<0.001^***^
Section 11 – places to go	Video time	0.00	0.00	1.24	0.414
	Child age	0.00	0.00	2.21	0.095
	Caregiver income	−0.00	0.00	−0.31	0.909
	Noun vocab (sect. 2–12)	0.01	0.00	3.58	<0.002^**^
**Section 12 – people**	**Video time**	**0.02**	**0.01**	**2.68**	**0.034** ^*^
	Child age	−0.02	0.01	−1.87	0.160
	Caregiver income	0.00	0.01	0.08	0.933
	Noun vocab (sect. 2–12)	−0.06	0.01	−6.73	<0.001^***^

*Note*: *p* values adjusted for multiple comparisons based on Benjamini‐Hochberg.

Significant video time effects are bolded.

^*^ *p* < 0.05, ^**^ *p* < 0.01, ^***^ *p* < 0.001.

### 3.2 Digital Media and Shape‐ and Material‐Based Words

Second, we examined associations between video watching and the proportion of nouns a child produced that named objects with shape‐based (e.g., ball, cup) versus material‐based (e.g., chalk, applesauce) category organization, controlling for child age, caregiver income, and total noun vocabulary. Video time did not predict either vocabulary class. See Table 3.^1^

**TABLE 3:** Regression analyses predicting shape and material vocabulary knowledge from video time controlling for overall vocabulary, child age, and caregiver income.

Model	Predictor	*Β*	Se	*t*	*p_adjusted_*
Proportion of shape‐based nouns	Video time	−0.00	0.01	−0.59	0.661
	Child age	0.01	0.01	0.66	0.661
	Caregiver income	0.01	0.01	1.31	0.381
	Noun vocab (2–12)	−0.03	0.01	−3.60	<0.003^**^
Proportion of material‐based nouns	Video time	−0.01	0.00	−2.02	0.118
	Child age	−0.00	0.00	−0.56	0.661
	Caregiver income	0.00	0.00	0.23	0.822
	Noun vocab	0.01	0.00	2.53	0.047^*^

*Note*: *p* values adjusted for multiple comparisons based on Benjamini‐Hochberg.

^*^ *p* < 0.05, ^**^ *p* < 0.01.

## 4 Discussion

We hypothesized that high rates of video/TV viewing would limit children's learning for specific types of words. This was due to two reasons: (1) use of videos/TV decreases children's opportunities for face‐to‐face social interactions (and thus learning of words that require social reference), and (2) videos/TV present content in ways that limit multi‐modal exploration that highlights specific features. The results suggest that even after controlling for a child's age, caregiver income, and noun vocabulary size, differences in children's video watching are associated with differences in the types of words children know. Specifically, children with higher rates of video exposure tend to have a higher proportion of people and furniture words and lower amounts of body part words.

Although a lower proportion of body parts words (e.g., arm, nose, ear) is consistent with the literature, the *higher* proportion of people words (e.g., mom, friend, girl) was unexpected because more digital media time tends to correlate with fewer face‐to‐face interactions (Brushe et al. 2024; Christakis et al. 2009) and thus, opportunities to learn such words. This could be due to two reasons: first, if caregivers are following the American Academy of Pediatrics recommendations to jointly engage in media use with their child (AAP Council on Communications and Media 2016), children may still maintain opportunities to learn words for social partners. In the current sample, however, exploratory analyses found no effect of joint engagement on vocabulary, and overall joint use of videos/TV was low—most families viewed videos jointly only half the time (*M* = 3.64 on a 5‐pt scale from “never” to “always”). Second, it is possible that children learned more people words because the content of the shows gives them an opportunity to do so. We know children more readily engage with and learn from video content that centers familiar characters (Lauricella et al. 2011; Richards and Calvert 2017). Exploratory analysis recorded the presence of such characters or people in the summaries of videos, the current sample reported and found approximately 64.5% of videos included people words (see Supporting Information). Thus, children may have increased attention to such shows due to the formation of parasocial relationships with media characters and increased pseudo‐interactions with those characters (Richards and Calvert 2017), aiding in learning people's words. In contrast, body part words are non‐existent in video content (1.1% of the videos in the current sample).

It remains an open question, however, as to why children with higher rates of video watching also learned a high proportion of furniture/room words. Furniture was not a common topic in children's videos (6.8% of videos in this sample), but it is possible furniture and room names are present within the domestic, everyday scenes of children's shows. Alternatively, it is possible that caregivers name furniture or rooms when directing children to “sit on the couch” to watch television, or that high media users simply see more furniture as they spend more time inside. Because there currently are no validated metrics to assess the content of children's shows, and in‐depth data on home experiences during media use are limited, these hypotheses cannot be directly tested. Until we develop better measures for capturing linguistic content and contexts of digital media use at an individual child level, we cannot identify why there are differences in the types of words children acquire.

Though videos/TV might provide chances to learn some types of words (people, furniture), it cannot supplement physical touch and thus, words that require multimodal input would still be limited. In line with this, we find evidence for limited learning of body part words, which compose some of the earliest categories of a child's vocabulary (Borovsky et al. 2016b). We did not find evidence for differences in the proportions of shape‐ or material‐based words. Although exposure to some of these nouns may be diminished by high rates of media use (e.g., less time physically touching items or playing in a sandbox or using playdough), many of children's early nouns, especially those for naming material‐based non‐solid substances, refer to very frequent items like toys (e.g., cup, ball) or food words (e.g., bread, applesauce; Samuelson and Smith 1999). Because young children are still playing with toys (including a tablet they might hold) and eating food (often with their hands), they may still maintain opportunities to learn these words. Future work measuring the broader family media ecology and context of media use can help to dissect these possibilities (see Barr, Kirkorian, Coyne et al. 2024). Particularly critical will be longitudinal vocabulary composition effects.

Taken together, the results here further demonstrate the need for more precise metrics to quantify both the linguistic context of media use as well as the input from digital media, akin to corpus‐level work in storybooks (see, e.g., Montag et al. 2015). Currently, capturing conversations during media use is labor‐intensive and resource‐heavy (see Sundqvist et al. 2022). Moreover, most content analysis is limited to “educational” labels, which are imprecise (Hirsh‐Pasek et al. 2015; Meyer et al. 2021). Future direct comparisons of digital input to face‐to‐face input, particularly longitudinally, could further illuminate the mechanisms of the relationship between media and language growth during these early years. However, without tools for assessing digital media content in conjunction with real‐time interactions during media use over time, we cannot dissect specific mechanisms for decreasing vocabulary or changes in specific vocabulary composition.

Most importantly, the current work demonstrates that the impact of digital media on children's language development is nuanced and may differentially affect children's language outcomes. If, as a field, we aim to identify how children's vocabulary emerges and subsequently, who may not be learning words as expected, we must consider children's digital media exposure and what it can support in vocabulary, but also what types of vocabulary it hinders.

## Author Contributions

**Sarah C. Kucker**: conceptualization, data curation, formal analysis, funding acquisition, investigation, methodology, project administration, resources, supervision, validation, visualization, writing – original draft, and writing – review and editing. **Rachel F. Barr**: funding acquisition, methodology, resources, and writing – review and editing. **Lynn K. Perry**: conceptualization, formal analysis, funding acquisition, methodology, visualization, and writing – review and editing.

## Funding

This study was supported by Eunice Kennedy Shriver National Institute of Child Health and Human Development, Grant/Award Number to SCK: R15HD101841.

## Ethics Statement

The current study was approved by the Oklahoma State University and Southern Methodist University IRBs. All individuals provided informed consent prior to participating.

## Conflicts of Interest

The authors have no conflicts of interest

## Supporting information




**Supporting File 1**: desc70091‐sup‐0001‐SuppMat.docx

## Data Availability

The data that support the findings of this study are available on the Open Science Framework: https://osf.io/zyr4f/?view_only=d5b0e7bd0f004b3ea821ac78a55deb56.

## Endnotes

1 The initial preregistered analysis used shape‐residuals instead of proportions, but proportions were used here to be consistent with the semantic class analysis. Analyses using the residuals found identical results and are reported in the Supporting Information.
