# Supplementary material for: Videos and Vocabulary: How Digital Media Use Impacts the Types of Words Children Know
Source: Dev Sci. 2025 Nov 10;29(1):e70091. doi: 10.1111/desc.70091 (PMC12602769; doi:10.1111/desc.70091)
Supplement: Supplementary file 1 — Supporting File 1: desc70091‐sup‐0001‐SuppMat.docx [file DESC-29-e70091-s001.docx]

**Videos and vocabulary: How digital media use impacts the types of words children know**

Sarah C. Kucker, Rachel F. Barr, & Lynn K. Perry

**Supporting Information**

**Preregistered analysis**

The original pre-registration including using shape residual scores in the final analysis instead of proportions. The current manuscript used proportions instead in order to align with the semantic category analyses (which were not pre-registered) and to make the output more interpretable. Using shape and material residuals instead, however, resulted in identical results as noted in Table S1, below.

**Table S1.** **Regression analyses predicting shape and material vocabulary residuals from video time controlling for overall vocabulary, child age, and caregiver income.**

| **Model** | **Predictor** | **Β** | **Se** | **t** | **P_adjusted_** |
| --- | --- | --- | --- | --- | --- |
| Shape residual score | Video Time | -.00 | .01 | -.60 | .777 |
|  | Child Age | .01 | .01 | .65 | .777 |
|  | Caregiver Income | .01 | .01 | 1.31 | .768 |
|  | Noun Vocab (2-12) | -.01 | .01 | -.66 | .777 |
| Material residual score | Video Time | -.01 | .00 | -2.01 | .359 |
|  | Child Age | -.00 | .00 | -.55 | .777 |
|  | Caregiver Income | .00 | .00 | .23 | .858 |
|  | Noun Vocab | .01 | .00 | .18 | .858 |

Note. P-values adjusted for multiple comparisons based on Benjamini-Hochberg

**Exploratory pilot data on content**

Caregivers in the current sample were asked to report the top three videos/TV shows/movies that their child watches. Of the 451 unique videos reported, 355 were able to be coded across all families who participated. Video titles that were vague (e.g. “YouTube” or “cartoons”), or not commercially available (e.g. “home videos of our family”) were not coded. The publicly available summary of each video from IMDb were saved to be scored; if the video was not available on IMDb, the summary from YouTube, or the host video’s website was used. Coders rated each video for the presence or absence of words related to each semantic vocabulary category. See Table S2.

**Table S2.** *Exploratory media content*

| **Word Category** | **Number of videos with content in category** | **Proportion of videos with content in category** |
| --- | --- | --- |
| **Sounds Effects** | 22 | 0.062 |
| **Animal Names** | 155 | 0.437 |
| **Vehicles/ Transportation** | 57 | 0.161 |
| **Toys** | 57 | 0.161 |
| **Food & Drink** | 24 | 0.068 |
| **Clothing/ Accessories** | 13 | 0.037 |
| **Body parts** | 4 | 0.011 |
| **Small Household Items** | 29 | 0.082 |
| **Furniture & Rooms** | 24 | 0.068 |
| **Outside Things** | 62 | 0.175 |
| **Places to Go** | 94 | 0.265 |
| **People** | 229 | 0.645 |
